# Symptom Distress and Quality of Life after Stereotactic Radiosurgery in Patients with Pituitary Tumors: A Questionnaire Survey

**DOI:** 10.1371/journal.pone.0088460

**Published:** 2014-02-05

**Authors:** Ching-Ju Yang, Guey-Shiun Huang, Fu-Ren Xiao, Meei-Fang Lou

**Affiliations:** 1 Department of Nursing, College of Medicine, National Taiwan University, Taipei, Taiwan; 2 Division of Neurosurgery, National Taiwan University Hospital, Taipei, Taiwan; Federal University of Rio de Janeiro, Brazil

## Abstract

**Background:**

Stereotactic radiosurgery (SRS) is a common treatment for recurrent or residual pituitary adenomas. The persistence of symptoms and treatment related complications may impair the patient’s quality of life (QOL).

**Purpose:**

The purpose of this study was to examine symptom distress, QOL, and the relationship between them among patients with pituitary tumors who had undergone SRS.

**Methods:**

This study used a cross-sectional design and purposive sampling. We enrolled patients diagnosed with pituitary tumors who had undergone SRS. Data were collected at the CyberKnife Center at a medical center in Northern Taiwan in 2012. A questionnaire survey was used for data collection. Our questionnaire consisted of 3 parts the Pituitary Tumor Symptom Distress Questionnaire, the World Health Organization Quality of Life Instrument Short-Form (WHOQOL-BREF), and a demographic questionnaire.

**Results:**

Sixty patients were enrolled in the study. The most common symptoms reported by patients after SRS were memory loss, fatigue, blurred vision, headache, sleep problems, and altered libido. The highest and lowest scores for QOL were in the environmental and psychological domains, respectively. Age was positively correlated with general health and the psychological domains. Level of symptom distress was negatively correlated with overall QOL, general health, physical health, and the psychological and social relationships domains. The scores in the psychological and environmental domains were higher in males than in females. Patients with ≤6 symptoms had better overall QOL, general health, physical health, and psychological and social relationships than those with >6 symptoms.

**Conclusion:**

Symptom distress can affect different aspects of patient QOL. Levels of symptom distress, number of symptoms, age, and gender were variables significantly correlated with patient QOL. These results may be utilized by healthcare personnel to design educational and targeted interventional programs for symptom management to improve patient QOL.

## Introduction

Pituitary tumors is accounting for 10%–15% of primary intracranial tumors. Apart from the complications that can occur as a result of treatment, pituitary tumors exhibit a variety of symptoms. Numerous studies have indicated that even after medical, surgical, or radiation therapy, patients continue to experience symptom distress in the physical, mental, and social domains [1]–[2]. Ongoing symptom distress and treatment-related complications can affect quality of life (QOL) of patients on different levels [3]–[4].

Compared with surgical therapy, stereotactic radiosurgery (SRS) is an advancement in the treatment of pituitary tumors. However, SRS cannot rapidly repair neurological symptoms or decrease hormone secretion and is mainly performed for recurrent or residual pituitary tumors [5]. Following SRS, the tumor control rate is reported between 83.3% and 100% [6]–[8]. Previous studies have reported that the hormone level recovery rate after SRS is 28.2%–35.2% and the average recovery time is 36.2±24 months [9]–[10]. Less than 10% of patients developed new visual acuity or field deficits after radiation therapy [6], [8]. Pituitary hypofunction following SRS was 1.7%–38%, increasing as time elapsed following treatment [8]–[10].

Symptoms have been defined as an individual’s perception of an abnormal physical, emotional or cognitive state and described as subjective, experienced, unpleasant and distressing [11]–[12]. Even after medical, surgical, or radiation therapy, patients continue to experience symptom distress in the physical, mental, and social domains. Therefore, it is important to evaluate a patient’s perception of the occurrence of symptoms and associated distress after SRS. Baird *et al.* examined patients with pituitary tumors who had undergone medical, surgical, or radiation therapy and found that 23% reported fatigue as the mostdistressing symptom. Sleep problems, memory loss, and altered libido were among other major concerns of patients [1]. Wu noted that the 5 main symptoms experienced by patients with pituitary tumors following surgery were fatigue (65.6%), sleep problems (50.0%), headache (34.4%), weight gain (32.2%), and blurred vision (28.9%) [13]. Cognitive impairment is a common complication in patients with pituitary tumors who have undergone surgery and radiation therapy [14]. Noad *et al.* examined patients with pituitary tumors who had undergone surgery and those who had undergone combined surgery and radiation therapy. Their findings showed that patients who had undergone additional radiation therapy showed significantly lower execution functions than those who had only undergone surgery. Overall, regardless of whether additional radiation therapy was administered, 27% of patients with pituitary tumors experienced visual memory impairment and 20% showed immediate memory impairment [15].

Ongoing symptom distress and treatment-related complications can affect QOL of patients. These patients might develop physical, psychological and social problems pertaining to their new lifestyle. Relevant studies on QOL of patients with pituitary tumors have indicated that compared with a healthy population, QOL was significantly lower on levels such as physiological functions, emotions, social interaction, pain, sleep, fatigue, anxiety, and depression [3], [16]–[17]. Moreover, in contrast to patients with nonfunctional pituitary tumors, those with functional pituitary tumors experienced higher rates of pain and physiological function impairment [17]. With regard to personal characteristics, female patients experienced a lower QOL, particularly concerning role limitations resulting from physical or emotional problems. Age and the physiological functions governing QOL showed a significant negative correlation [3], [17]–[18]. Research indicates that symptom distress shows a significant negative correlation with QOL in all domains [4], [19].

The purpose of this study was to examine symptom distress, QOL, and the relationship between them among patients with pituitary tumors who had undergone SRS.

## Methods

### Ethical Considerations

This study was reviewed and approved by the Research Ethics Committee (No. 201112063RIC) of National Taiwan University Hospital, where it took place. Participants were explicitly informed that the data produced in the survey would be confidential, would not affect their treatment, and would be used only for academic research purpose. Written informed consent was obtained from all participants.

### Design

This study was a cross-sectional survey research design of patients who had undergone SRS at a CyberKnife Center in a medical center in Northern Taiwan.

### Sample

Patients who underwent SRS at the center from its establishment in February 2008 to December 2011 were included. The inclusion criteria were as follows: (1) patients diagnosed with pituitary tumors; (2) patients who had undergone a course of SRS at least 3 months before commencement of this research; and (3) adult patients who were conscious, and able to communicate in Mandarin or Taiwanese. The sample size was calculated on the basis of results from a literature review. The function used to estimate the sample size was adopted from Lindsay *et al*. [4]. The correlation coefficients (r) for symptom distress and QOL were −0.5 (physical health domain) and −0.6 (psychological health domain), respectively [4]. The two-tailed alpha level was set at 0.05, and the desired power was set at 0.9. We used G-power 3.0 software to calculate the required sample size [20]. When α = 0.05, β = 0.1 (power = 1 − β = 0.9), correlation coefficient r = 0.5 was entered into the correlation model for estimation, *a minimum* of 34 patients were needed in the study.

### Data Collection

Data collection was conducted using a questionnaire survey. The research data were gathered between February 2012 and April 2012. The case manager at the CyberKnife Center contacted patients via telephone to ascertain their willingness to participate in this research. At the outpatient clinic or scheduled at another location and time by patient’s convenience, the researchers explained the patients regarding the purpose and process of the research and the procedure regarding obtaining data via questionnaires, after which informed written consent was obtained. Data collection required 30–40 minutes for each patient. If patients requested questionnaires via post services, questionnaires were distributed along with a return envelope. If there was no reply within 2 weeks, patients were reminded about completing and returning the questionnaire by telephone.

Our questionnaire consisted of the following three parts:

**Demographic questionnaire.** This questionnaire included two parts: personal characteristics and disease-related information. The personal characteristics part was completed by patients, and it included information such as age and gender. Disease-related information was obtained from medical records, and it included data such as number of medications used, radiation dosage received, target volume, perisellar invasion, history of surgical procedures prior to SRS, pituitary tumor type, time after undergoing SRS, and reasons for undergoing radiosurgery.

**Pituitary tumor symptom distress questionnaire.** This questionnaire is based on a list of complaints associated with pituitary tumors. It was initially developed by Baird *et al.* [1] and was revised by Wu [13]. The instrument used in this study was revised by the investigators based on the literature and clinical experience. The questionnaire includes questions regarding 25 common symptoms observed in patients with pituitary tumors, and the patients evaluated the presence of these symptoms and levels of distress on the basis of their subjective perception. Symptom prevalence was calculated on the basis of whether the patient currently exhibited a certain symptom. Levels of symptom distress were evaluated using a 5-point Likert scale (distress levels 0, none; 1, mild; 2, moderate; 3, severe; and 4, extreme). Overall scores ranged between 0 and 100, with higher scores indicating higher levels of symptom distress. The content validity of this questionnaire was established by evaluation of the relevance and clarity of the content of each item by 3 experts in this field. This study had an overall scale internal consistency reliability (Cronbach’s α) of 0.90.

**World Health Organization Quality of Life instrument short-form (WHOQOL-BREF).** This study used the WHOQOL-BREF Taiwan version comprising 28 items including overall QOL (one item), general health (one item), and the 4 domains regarding physical health (7 items), psychological (6 items), social relationships (4 items), and environmental factors (9 items). Questionnaire items use a 5-point Likert scale ranging from 1 to 5, with higher scores indicating better QOL [21]. We used the WHOQOL–BREF because it is a well-known and widely used instrument in QOL research. This scale possesses high reliability and validity, as shown in numerous studies and groups. All domains have internal consistency reliabilities (Cronbach’s α) of 0.70–0.77. Overall questionnaire internal consistency reliability (Cronbach’s α) is 0.91, and the interdomain correlation is 0.51–0.64. Factor analysis results showed that 73% variance accounted for the 4 domains [22]. This study had internal consistency reliabilities (Cronbach’s α) of 0.71–0.91.

### Data Analysis

This research used SPSS 18.0 for Windows/PC packaged software (Chicago, IL, USA). A *p* value of <0.05 was considered significant, and Spearman’s rank correlation, the Mann–Whitney U-test, and the Kruskal–Wallis one-way analysis of variance by rank were used as inferential statistical methods to examine the correlations and differences among personal characteristics, symptom distress, and QOL.

## Results

### Patient Characteristics

Between February 2008 and December 2011, 100 patients with pituitary tumors underwent SRS at the CyberKnife Center. Among these patients, 25 patients refused to participate in this research, nine could not be contacted, three experienced changes in consciousness or orientation, two had died, and one had returned an incompletely filled questionnaire. We were able to successfully obtain data from 60 patients. Apart from the fact that nonparticipants were of a significantly higher age, there were no significant differences in regard to personal characteristics and tumor type between them and the participants.

Thirty-two patients were male (53.3%) and the mean age of the total patient population was 51.17 years (range, 24–81 years). Twenty-five (41.7%) patients had undergone SRS <2 years prior to conducting this research and 35 (58.3%) had undergone SRS ≥2 years prior to conducting this research. Twenty-nine (48.3%) patients had undergone SRS because of residual tumor and 27 (45.0%) because of recurrence. Fifty-six patients (93.3%) had undergone other surgical treatment prior to SRS. Twenty-six patients (43.3%) had nonfunctional pituitary tumors, and 25 (41.7%) had functional pituitary tumors; >60% patients aged ≤51 years had functional pituitary tumors, whereas approximately 70% patients aged >51 years had non-functional pituitary tumors. Twenty-eight (46.7%) had pituitary tumors showing perisellar invasion. Thirty-three (55%) patients were receiving pituitary tumor-related medication (primarily hormonal and antiepileptics) over the period when this research was conducted.

### Symptom Distress after SRS in Patients with Pituitary Tumors

The average number of symptom recorded was 5.95±5.05 (range, 0–19), and the average total score for the levels of symptom distress was 7.62±9.96 (range, 0–55). When including only those patients experiencing a particular symptom, the mean ratings on symptom distress for each item was 1.35±0.58, denoting mild to moderate levels. The most common symptoms reported by patients were memory loss (31, 51.6%), fatigue (28, 46.7%), blurred vision (23, 38.4%), headache (20, 33.3%), sleep problems (19, 31.7%), and altered libido (19, 31.7%) (Table 1).

**Table 1:** Distribution of symptom distress after Stereotactic Radiosurgery in patients with pituitary tumors (*n* = 60).

Variable	No.	%	Mean ± SD (range)
**Number of symptoms**			5.95±5.05 (19)
**Levels of symptom** **distress**			7.62±9.96 (55)
**Most common** **symptoms**			
Memory loss	31	51.6	1.26±1.15
Fatigue	28	46.7	1.13±0.90
Blurred vision	23	38.4	1.22±0.95
Headaches	20	33.3	1.35±1.04
Sleep problem	19	31.7	1.74±1.10
Altered libido	19	31.7	0.84±1.26

SD: standard deviation.

### Quality of Life after SRS

The highest score among the 4 domains of QOL was for the environmental domain (14.74±1.79), followed by physical health (14.10±2.64), social relationships (13.85±2.14), and psychological (13.51±2.61) domains (Table 2). The lowest scoring items among the QOL domains were positive feelings (3.03±0.74, psychological domain), followed by sexual activity (3.07±0.78, social relationships domain), and vitality and fatigue (3.13±0.79, physical health domain), indicating a moderate deterioration in QOL. QOL-correlated factor analysis indicated that age and general health (Spearman’s ρ = 0.51, *p = *0.00) and psychological domains (Spearman’s ρ = 0.33, *p = *0.01) showed a significant positive correlation. There was a significant negative correlation between the levels of symptom distress and physical health, psychological, and social relationships domain scores; overall QOL; and general health (Spearman’s ρ = −0.43 to −0.69, *p = *0.00). However, no significant correlation was found between the levels of symptom distress and the environmental domain scores (Table 3). The psychological (Z = −2.33, *p* = 0.02) and environmental domains scores (Z = −2.08, *p = *0.04) in male patients were significantly higher than those in female patients. Patients with a total of ≤6 symptoms had significantly higher scores for physical health, psychological, and social relationships domains; overall QOL; and general health than those with >6 symptoms (Table 4).

**Table 2:** Descriptive analysis of patients’ quality of life (QOL) scores (*n* = 60).

Domain	No. of items	Mean ± SD (range)
Overall quality of life	1	3.43±0.77 (4.00)
General health	1	2.87±1.05 (4.00)
Physical health	7	14.10±2.64 (14.29)
Psychological	6	13.51±2.61 (12.00)
Social relationships	4	13.85±2.14 (11.00)
Environmental factors	9	14.74±1.79 (8.44)

SD: standard deviation.

**Table 3:** Factors correlating with domains of quality of life (QOL) as well as overall QOL (*n = *60).

Variable	Overall QOL	General Health	Physical Health	Psychological	Social Relationships	Environmental
	ρ^a^ (*p*)	ρ^a^ (*p*)	ρ^a^ (*p*)	ρ^a^ (*p*)	ρ^a^ (*p*)	ρ^a^ (*p*)
Age	0.18 (0.17)	0.51*(0.00)	0.18 (0.17)	0.33*(0.01)	0.11 (0.11)	0.18 (0.18)
Number of medication	0.16 (0.22)	−0.14 (0.30)	0.05 (0.72)	−0.04 (0.76)	−0.19 (0.14)	0.21 (0.11)
Radiation dose	0.01 (0.95)	−0.04 (0.78)	0.08 (0.54)	−0.08 (0.54)	−0.12 (0.36)	−0.12 (0.35)
Target volume	0.02 (0.88)	0.08 (0.56)	0.01 (0.97)	0.11 (0.39)	0.07 (0.60)	0.11 (0.42)
Levels of symptom distress	−0.57*(0.00)	−0.69*(0.00)	−0.55*(0.00)	−0.54*(0.00)	−0.43*(0.00)	−0.12 (0.42)

*p<0.05;

a Spearman’s rank correlation coefficient.

**Table 4:** Differences in personal characteristics, levels of symptom distress, and quality of life (QOL) (*n* = 60).

Variable		Overall		General		Physical		Psychological		Social		Environmental	
		QOL		Health		Health				Relationships			
	No.	Mean	χ^2a^	Mean	χ^2a^	Mean	χ^2a^	Mean	χ^2a^	Mean	χ^2a^	Mean	χ^2a^
		Rank	(*p*)	Rank	(*p*)	Rank	(*p*)	Rank	(*p*)	Rank	(*p*)	Rank	(*p*)
**Gerder**			0.82		−1.17		−0.85		−2.33*		−0.82		−2.08*
Male	32	32.08	(0.41)	32.86	(0.24)	32.28	(0.40)	35.38	(0.02)	32.20	(0.41)	38.46	(0.04)
Female	28	28.70		27.80		28.46		24.93		28.55		25.52	
**Perisellar invasion**			−0.64		−0.43		−0.97		−0.75		−0.90		−0.70
Yes	28	31.91	(0.52)	29.50	(0.67)	32.82	(0.33)	32.30	(0.45)	32.63	(0.37)	32.18	(0.48)
No	32	29.27		31.38		28.47		28.92		28.64		29.03	
**Under gone** **surgery**			−1.20		−0.26		−0.88		−1.83		−0.59		−0.67
Yes	56	31.16	(0.29)	30.65	(0.81)	31.03	(0.40)	31.60	(0.07)	30.85	(0.58)	30.09	(0.52)
No	4	21.25		28.38		23.13		15.13		26.63		36.25	
**Time after SRS**			−1.10		−1.68		−0.79		−1.30		−0.65		−0.54
<2 years	25	33.18	(0.27)	34.80	(0.09)	32.58	(0.43)	33.94	(0.20)	32.20	(0.52)	31.94	(0.59)
≥2 years	35	28.59		27.43		29.91		28.04		29.29		29.47	
**Number of Symptoms**			−4.06*		−4.69*		−5.33*		−4.69*		−3.11*		−1.40
≤6	35	37.57	(0.00)	39.14	(0.00)	40.60	(0.00)	39.39	(0.00)	36.34	(0.00)	33.14	(0.16)
>6	25	20.60		18.40		16.36		18.06		22.32		26.80	

*p<.05,

a Mann–Whitney U-test.

## Discussion

This research shows that the symptom most frequently reported by patients with pituitary tumors after SRS was memory loss. The highest and lowest scores for QOL were in the environmental and psychological domains, respectively. QOL showed correlations with age, gender, levels of symptom distress, and total number of symptoms.

### Symptom Distress after SRS

This study found that the levels of symptom distress for patients were 7.62±9.96 and the mean number of symptoms reported was 5.95±5.05. The maximum distress level score for the 25 symptoms surveyed in this study was 100, indicating that patients with pituitary tumors did not score highly for symptom distress after undergoing SRS, possibly because of the differences in pituitary tumor type and diversity of symptoms. For example, patients with functional and nonfunctional pituitary tumor showed significant differences in the number of symptoms and symptom distress levels. In addition, in this study, 93.3% of patients had undergone surgical treatment before undergoing SRS, indicating that symptom distress may have improved compared with the results before surgery, resulting in a lower symptom distress level after SRS than expected, with some symptom distress being attributed to the primary surgery.

This study shows that common symptoms reported after SRS include memory loss, fatigue, blurred vision, headache, sleep problems, and altered libido. Wu studied patients with pituitary tumors who had undergone surgery and observed common postoperative symptoms including fatigue, sleep problems, headache, weight gain, blurred vision, and altered libido [13]. Both these studies show that regardless of undergoing surgery or radiation therapy, common symptoms experienced by such patients include fatigue, sleep problems, headache, blurred vision, and altered libido. According to the literature, decreased functions of the adrenals, thyroid, and gonadal glands have been shown to cause fatigue in patients [23]–[25]. Altered libido may be related to the type of pituitary tumor, menopause, or androgen deficiency [26]–[28]. Sleep problems may cause fatigue [29], and 31.7% of patients in this study reported sleep problems. Further analysis was performed on the relationship between sleep problems and fatigue within this study, showing a significant correlation. Apart from these symptoms, previous research has shown that the most common symptom for which patients with pituitary tumors are hospitalized is the impairment of vision [30]. Furthermore, 68.8%–70.0% of patients experience headache [2], [31]–[32]. The results of our study support those of Wu in that several patients with pituitary tumors continue to experience symptom distress such as headache and blurred vision, regardless of previous surgery or SRS [13].

Our study and that by Wu investigated the postoperative symptom distress after SRS and surgery, respectively, in patients with pituitary tumors [13]. However, although Wu did not survey the symptom of memory loss, our study shows that after undergoing SRS, >50% of patients suffered memory loss. Previous studies have shown that cognitive impairment or memory loss are common symptoms experienced by patients with pituitary tumors after undergoing surgery or radiation therapy [1], [14]. Noad *et al.* examined patients with pituitary tumors who had undergone surgery and those who had undergone combined surgery and radiation therapy. Overall, regardless of whether additional radiation therapy had been administered, 27% of patients experienced visual memory impairment and 20% experienced immediate memory impairment. However, the patients in our study showed a higher rate of memory loss than those examined by Noad *et al.*, possibly because our research was based on patients’ subjective perceptions [15]. This may have been the cause of the observed differences. Patients’ experiences and perceptions of memory loss vary, and the large standard deviations suggest that some individuals experience more severe problems than others. Pituitary tumors disrupt the hypothalamic–pituitary-target gland axis and cause dysfunction in physical and psychological domains. Altered interplay among the components of the hippocampus-hypothalamic-pituitary axis is related to cognitive impairment. However, patients with some types of pituitary tumors experienced a higher degree of cognitive impairment. For example, memory and executive dysfunction are often observed in patients with pituitary adenoma with excessive corticotropin secretion [33]. In a review by Taphoorn and Klein [34] on cognitive deficits in adult patients with brain tumors, it was reported that cognitive impairments were due to the combinations of tumor, tumor-related epilepsy, and treatment effects (surgery, radiotherapy, antiepileptics, chemotherapy, or corticosteroids) [34]. Although patients in this study had the same diagnosis (pituitary tumor) and received the same treatment (SRS), the tumor types varied. The small sample size does not allow for further subgroup analysis. Such an analysis in a larger sample size is recommended to determine the actual significance of cognitive impairment. Other possible contributing effects include age and sleep problems. Episodic memory is known to decrease with age [35]; 55% of the patients in this study sample were >50 years old, and the high average age of the patient population in this study may be a confounding factor. Sleep enhances memory consolidation [36], and 31.7% of patients in this study had sleep problems. We performed an analysis to assess the relationship between sleep problems and memory and found a significant correlation. Therefore, sleep problems may contribute to memory loss. Future research in an age-matched healthy control group would be useful to validate the significance of patient responses.

### Quality of Life after SRS

Among the 4 domains of QOL, the patients in this study scored lowest in the psychological domain, with scores for most items in this domain being at a moderate level. Similar findings were reported in a study by Skevington and McCrate, who investigated patients with 27 diseases of varying status, comparing them with healthy populations (*n* = 4,628) in the UK, using WHOQOL-BREF to evaluate QOL [37]. In that study, the endocrine-related group (*n* = 524) scored lowest in the psychological domain of QOL. It is possible that >90% of patients in that study had undergone surgical therapy and approximately 50% underwent radiosurgery to remove residual tumors, with physical health domain scores being improved consequently. However, some patients required continuous medication for hormonal replacement or supplementation [37]. Different types of pituitary tumors show different responses to SRS. The effects of SRS require long-term patient follow-up because SRS cannot rapidly repair neurological symptoms. In addition, the probability of postoperative hypopituitarism increases over time [7], [9] and unknown or uncertain factors relating to physical function may lead to patients’ sense of uncertainty, anxiety, and depression [38]–[39], thereby resulting in a lower score in the psycological domain of QOL. Our research did not obtain data concerning patients’ sense of uncertainty, anxiety, and depression, and future research examining these factors is required.

Previous resarch has shown that all levels of symptom distress show a significant negative correlation with QOL [4], [19]. Our study supports these findings by identifying a significant negative correlation between the levels of symptom distress and QOL, including overall QOL and the general health, physical health, psychological, and social relationships domains. Regarding age and gender, previous studies have shown that female patients experience lower QOL, especially with regard to role limitations resulting from physical or emotional problems, with the relationship between age and the physical health domain of QOL showing a significant negative correlation [3], [17]–[18]. Our research also found that the scores for the psychological domain of QOL in female patients was significantly lower than those in male patients, although there was no significant difference between the sexes with regard to physical health domain scores. Our study showed that age and the general health and psychological domains of QOL had a significant positive correlation, thereby indicating that older patients have better overall health and improved psychological status, potentially because the mean age of patients in this study was 51.10 years. More than 60% of patients aged ≤51 years had functional pituitary tumors, whereas approximately 70% of patients >51 years had nonfunctional pituitary tumors, i.e., older patients tended to have nonfunctional tumors. Previous research has shown that in contrast to patients with nonfunctional pituitary tumors, those with functional pituitary tumors experience greater impairment of physiological function and pain [17]. The differences in distribution of pituitary tumor types may be the cause of variations in research results.

In this study, we found that age, gender, level of symptom distress, and total number of symptoms influenced the QOL of patients with pituitary tumor. Effective symptom management strategies may improve QOL. For example, physical activity programs have been developed, and meta-analysis of the results revealed that such programs improved both physical function and psychological function (decreased levels of fatigue and depression) [40]. Narrative synthesis study findings related to exercise programs also reported positive effects on cognition [41]. Recent decades have observed an increasing number of clinical trials involving cognitive training interventions for the elderly. Engelberts and colleagues reported that a cognitive training program improved cognitive function and QOL in patients with focal seizures [42]. Studies that have assessed the effects of cognitive training on memory in elderly persons also reported positive effects [43]–[45]. Through education, referral, or implementation of targeted symptom management strategies for patients, healthcare personnel may help patients better manage their symptoms and improve QOL.

### Limitations and Suggestions

The limitations of this study result from the use of a cross-sectional study design and the lack of a control group, which led to an inability to obtain information regarding either changes in symptom distress and patient QOL before and after SRS as well an inability to obtain the differences in symptom distress compared with healthy groups when incorporating or matching various age and gender groups. This study could only examine patients’ overall symptom distress and QOL at a particular point in time. Furthermore, symptom disturbance is a subjective measure, and our study lacked correlated objective measurements, such as blood hormone levels. Future research should apply prospective designs to follow up patients’ long-term symptom distress and altered QOL before and after SRS. In addition, both subjective and objective indicators can be used to monitor the changes in symptom disturbances pre- and post-SRS, and these data can be compared with healthy persons or a control group of patients undergoing simple surgery or with other brain tumor diagnoses. To better understand patient responses, a more homogeneous group, such as a subgroup of pituitary tumor patients, should be included in future research designs. Because of the relatively short time since the establishment of the CyberKnife Center from which cases were selected for this study, we could only collect information regarding patients who had undergone SRS within 4 years of our study period. The small sample size from a single center limits the generalizability of the results. Long-term follow-up with a larger sample size and the inclusion of other variables such as anxiety and depression is recommended for future studies. Symptom management and intervention should be implemented regarding the most common or disturbing symptoms for patients with pituitary tumors to facilitate improvement in their QOL.

### Conclusion

This study found that the most common symptom reported by patients with pituitary tumors after SRS was memory loss. The highest score among domains of QOL was in the environmental domain and the lowest was in the psychological domain. Correlated factors of QOL included age, gender, levels of symptom distress, and total number of symptoms. Age and gender are constant factors, although symptom distress can change dynamically according to the individual, social environment, stage of therapy, and disease progression. Examining QOL is not limited to understanding the current status of the patient; healthcare personnel should further develop interventions to effectively improve patient QOL.
